# Light-induced structural changes in a monomeric bacteriophytochrome

**DOI:** 10.1063/1.4961911

**Published:** 2016-08-29

**Authors:** Heikki Takala, Stephan Niebling, Oskar Berntsson, Alexander Björling, Heli Lehtivuori, Heikki Häkkänen, Matthijs Panman, Emil Gustavsson, Maria Hoernke, Gemma Newby, Federico Zontone, Michael Wulff, Andreas Menzel, Janne A. Ihalainen, Sebastian Westenhoff

**Affiliations:** 1 Department of Chemistry and Molecular Biology, University of Gothenburg, Gothenburg 40530, Sweden; 2 Nanoscience Center, Department of Biological and Environmental Sciences, University of Jyvaskyla, Jyväskylä 40014, Finland; 3 Faculty of Medicine, Anatomy, University of Helsinki, Helsinki 00014, Finland; 4 Nanoscience Center, Department of Physics, University of Jyvaskyla, Jyväskylä 40014, Finland; 5 BIOSS Centre for Biological Signalling Studies, Pharmaceutical Sciences, Albert-Ludwigs-Universität, Freiburg i. Br., Germany; 6 ESRF–The European Synchrotron Radiation Facility, CS40220, 38043 Grenoble Cedex 9, France; 7 Paul Scherrer Institut, 5232 Villigen PSI, Switzerland

## Abstract

Phytochromes sense red light in plants and various microorganism. Light absorption causes structural changes within the protein, which alter its biochemical activity. Bacterial phytochromes are dimeric proteins, but the functional relevance of this arrangement remains unclear. Here, we use time-resolved X-ray scattering to reveal the solution structural change of a monomeric variant of the photosensory core module of the phytochrome from *Deinococcus radiodurans.* The data reveal two motions, a bend and a twist of the PHY domain with respect to the chromophore-binding domains. Infrared spectroscopy shows the refolding of the PHY tongue. We conclude that a monomer of the phytochrome photosensory core is sufficient to perform the light-induced structural changes. This implies that allosteric cooperation with the other monomer is not needed for structural activation. The dimeric arrangement may instead be intrinsic to the biochemical output domains of bacterial phytochromes.

## I. INTRODUCTION

**FIG. 1.**
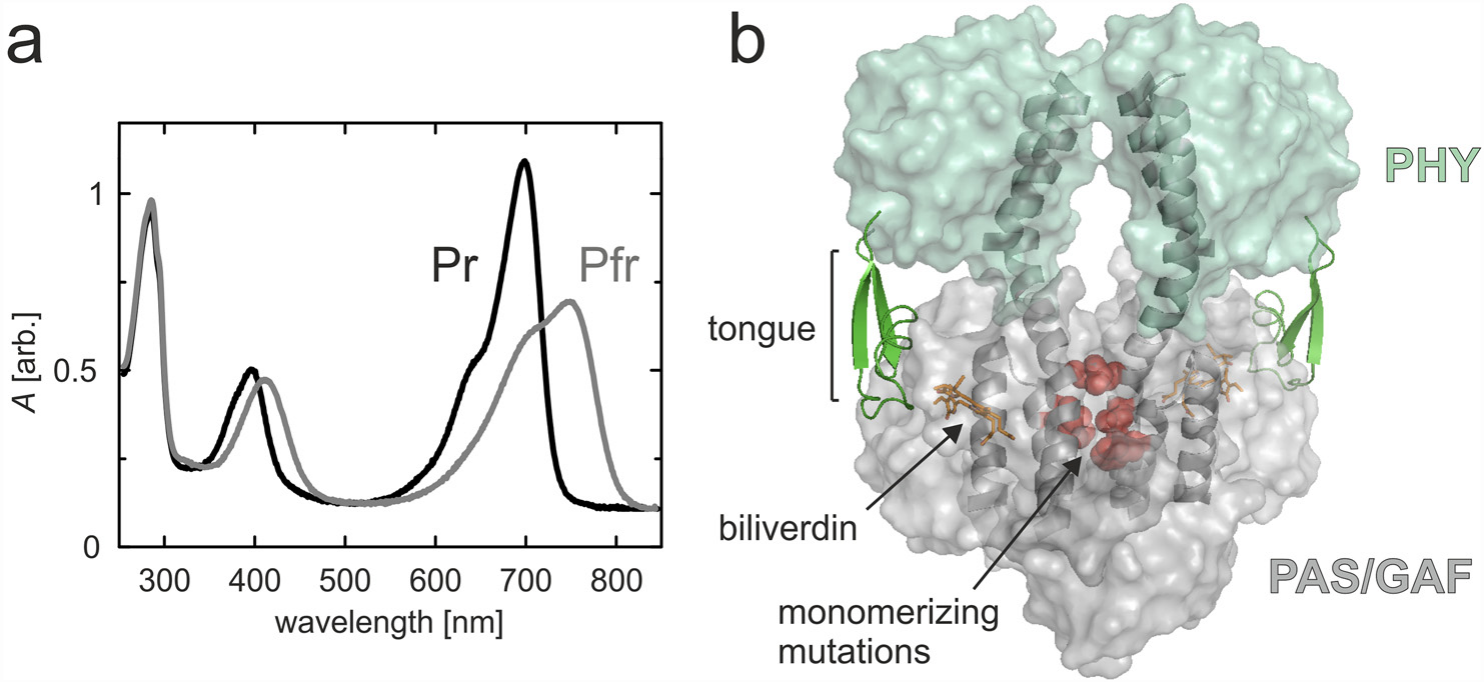
UV-Vis spectra and overview structure of the *D. radiodurans* fragments. (a) UV-Vis spectra of the PAS-GAF-PHY_mon_ in Pr and Pfr states. (b) Domain structure of the photosensory module fragment (PDB code 4Q0J^38^). PAS and GAF domains are shown in grey and the PHY domain in cyan. The tongue (*green*) is presented as cartoons, as are the scaffolding helices that form the dimerization interface. The biliverdin chromophore (*orange*) is presented as sticks, and the monomerizing mutations of three residues which inhibit interactions between the monomers are presented as red spheres.

Phytochromes are photoreceptors in plants and certain microorganism that sense red/far-red light and convert the signal to a biological response. Bacterial phytochromes often function as histidine kinases (HKs) in two-component signaling systems.^1^ Phytochromes bind a tetrapyrrole bilin chromophore, which is phytochromobilin (PΦB), phycocyanobilin (PCB), or biliverdin (BV) in plants, cyanobacteria, or bacteria, respectively. In response to incident light, they can switch between a red-light-absorbing “Pr” state and a far-red-light-absorbing “Pfr” state. In prototypical phytochromes, the Pr state is the resting state and the Pfr state is metastable (Fig. 1(a)).^1^

Phytochromes have a modular architecture with specific photosensory and signaling parts (Fig. 1(b)). The light-sensing photosensory core module consists of PAS (Per, Arnt, Sim) and GAF (cGMP phosphodiesterase, adenylate cyclase, FhlA) domains and a PHY (Phytochrome-specific GAF related) domain. The bilin chromophore is covalently linked to a conserved cysteine in either the PAS domain (bacteria) or the GAF domain (plants, cyanobacteria). The majority of chromophore-protein interactions are within the PAS and GAF domains, which are therefore collectively called the chromophore-binding domain. An important additional chromophore-related interaction is with a “hairpin,” “tongue,” or “arm” extension of the PHY domain. This motif extends from the bulk body of the PHY domain to the vicinity of the chromophore. The photosensory module (PAS-GAF-PHY) is followed by an output module, which is often a histidine kinase (HK) domain in bacteria and cyanobacteria but is more variable in plants.^2,3^

Incident red/far-red light causes a *Z*-to-*E* isomerization of the C15=C16 double bond in the chromophore.^4–7^ These changes are then relayed and amplified to the rest of the protein. It has been proposed that the PHY tongue refolds upon light absorption,^8,9^ and this has been verified by crystallography.^10^ The tongue adopts a β-turn-like conformation in the Pr state but an α-helix and coil in the Pfr state.^8,10–12^ Thereby, it switches between two highly conserved interaction patterns that include the PRxSF motif of the tongue and the DIP motif in the GAF domain.

We have previously shown how refolding of the PHY tongue in the *Deinococcus radiodurans* phytochrome is associated with the change in the relative orientation of the PHY domains. As a result, the sister PHY domains of the dimeric photosensory module (PAS-GAF-PHY) separate by several nanometers in the Pfr state, compared with the Pr state.^10^

Phytochromes are usually dimers. It is not understood if both monomers have to be photoactivated for output (de)activation. In bacteria, the function of the HK output domains probably requires interdimer interactions.^13^ It has also been speculated that allosteric photoactivation of the dimer is intrinsic to the generation of a structural signal in the photosensory core module.^14^ Thus, it is interesting to find out if the structural signal in the photosensory core appears after absorption into only one monomer of the dimer, or if both monomers have to be photoswitched, i.e., if they are allosterically coupled.

Monomeric variants of a phytochrome from *D. radiodurans* have been engineered by mutating residues F145, L311, and L314 in the dimer interface. They were used to create red fluorescent proteins.^15^ Monomeric phytochrome variants also have potential for applications based on light-controlled dimerization.^16^ Hence, it is important to find out if a phytochrome monomer can undergo the conformational responses to light without its dimerization partner.

Here, we study the light-induced conformational changes of the monomeric variant of the photosensory module from *D. radiodurans* (PAS-GAF-PHY_mon_).^16^ For this, we apply time-resolved X-ray solution scattering and Fourier transform infrared (FTIR) spectroscopy. We first describe the structural changes within the monomer as observed by the X-ray scattering data. The data reveal more structural details than what was observed for the dimeric phytochrome. We then describe the FTIR data, which are consistent with a decrease in β-sheet content and an increase in helicity. Finally, we discuss the results in terms of potential allostery and the role of the dimeric arrangement of phytochromes.

## II. EXPERIMENTAL PROCEDURES

### A. Cloning and protein purification

The expression plasmids for monomeric and dimeric fragments are described elsewhere.^16^ Protein expression and purification was as published elsewhere.^10,17,18^ The expression of phytochrome variants in the *Escerichia coli* strain BL21 DE3 was induced with 1 mM IPTG followed by inoculation at 26 °C for 18 h. The cells were then lysed with EmulsiFlex^®^, and the lysate was cleared by ultracentrifugation. An excess of solubilized biliverdin hydrochloride (Frontier Scientific) was added to the cell lysate, and the samples were incubated overnight on ice to allow holoprotein formation. All steps after biliverdin addition were conducted in dark. The His_6_-tagged phytochrome samples were first purified with NiNTA affinity purification using HisTrap™ columns (GE Healthcare), followed by size-exclusion chromatography (HiLoad™ 26/600 Superdex™ 200 pg, GE Healthcare) in running buffer (30 mM Tris, pH 8.0). The final samples were concentrated to 30 mg/ml (0.53 mM in the case of PAS-GAF-PHY_mon_) and flash-frozen. The phytochrome solutions were thawed and filtered with a 0.22 *μ*m centrifugal filter (Amicon) just before X-ray scattering measurements.

### B. Time-resolved wide-angle X-ray scattering

Millisecond time-resolved X-ray scattering was recorded at the Coherent Small-Angle X-ray Scattering (cSAXS) beamline at the Swiss Light Source as previously described.^19^ The station uses a rapid-readout detector to detect the time-evolution of the X-ray scattering signal after reaction initiation with a laser pulse.^20^ In brief, the sample (30 mg/ml) was pumped through a 1.0 mm quartz capillary between each data acquisition sequence. In each sequence, the Pr and Pfr states were prepared with a red, diode-pumped solid state laser (λ = 671 nm, 0.9 mJ mm^−2^, spot size 800 × 250 *μ*m, Altechna) and a far-red diode laser (λ = 789 nm, 12 mJ mm^−2^, 1300 × 1300 *μ*m, Thorlabs). While photoswitching the sample, the X-ray scattering (X-ray energy of 11.2 keV) was recorded simultaneously on two detectors, a Pilatus 2M and a Pilatus 300K-W. The detectors were read out at 25 Hz, with 35 ms for image acquisition and 5 ms for readout. The 2M detector covered the small-angle region (*q* = 0.05 nm^−1^ to *q* = 6.7 nm^−1^) and the 300 K detector recorded scattering at larger angles (*q* = 5.2 nm^−1^ to *q* = 27.5 nm^−1^). The overlap between the two ranges was used for scaling the two detector readouts. The energy of the red excitation laser pulse was set by controlling the opening time of a mechanical shutter to 5 ms. The turnover was estimated by titrating the laser power and measuring the amplitude of the difference scattering signal of the full-length dimeric phytochrome from *D. radiodurans* with details described in Ref. 21. Assuming that the monomeric variants studied here have the same excitation probability as the dimeric phytochrome,^21^ a turnover of 25%–40% from Pr to Pfr is estimated. The far-red laser pulse was adjusted to 50 ms, which was confirmed to achieve complete back-conversion as judged by the recorded difference scattering signals. Radiation damage was removed from the data by subtracting the average of the preceding and following acquisition without any optical lasers from the acquisition with laser flashes.^21^

Microsecond time-resolved X-ray scattering measurements were conducted at the beamline ID09b of the European Synchrotron Radiation Facility (ESRF) as described in Ref. 21. The sample (30 mg/ml, or 0.35 mM) was pumped (120 *μ*l/min) through a quartz capillary. A red nanosecond laser (λ = 660 nm, 1.4–3.5 mJ mm^−2^, spot size 200 × 1000 *μ*m) was focused to the capillary together with the overlapping far-red recovery laser (λ = 789 nm, 1.25 mJ mm^−2^, spot size 1500 × 1500 *μ*m, Thorlabs) and the X-ray pulses. The X-ray energy was 18 keV, and the X-ray pulse length was 2 *μ*s. 5-fold dilution of the sample did not affect the signal shape or kinetics (Fig. S2 in supplementary material).

For both experiments and following standard practice, the 2D readout from the detector was integrated in rings and normalized in the region of 14 nm^−1 ^< *q* < 16 nm^−1^. The heating signal was measured and subtracted from the data as before.^19^

### C. Model preparation and molecular dynamics simulation

Molecular Dynamics (MD) simulations were performed with GROMACS 4.5.5^22,23^ using the Charmm27 force field.^24^ Force-field parameters for the biliverdin chromophore in the Pr and Pfr state^25^ were used as described in Ref. 10.

Single chains from the starting Pr and Pfr structures from previous PAS-GAF-PHY simulations^10^ were used as templates for creating the starting structures. The three monomerizing mutations (F145S, L311E, and L314E) were applied accordingly to the model. The structures were energy-minimized with a deepest descent minimization (convergence criteria 500 000 steps or maximum force <2000 kJ mol^−1^ nm^−1^). Afterwards, both structures were solvated in a cubic box with periodic boundary conditions and a side length of 12 nm comprising the protein, with 54 000 (Pr simulation) and 66 000 (Pfr simulation) water molecules and Na^+^/Cl^−^ ions corresponding to a salt concentration of 0.1 M. The charge of the protein (-26) was neutralized by adding Na^+^ ions. After a steepest-descent minimization with the parameters listed above, two 100 ps equilibration MD runs were performed: The first one in the constant particle number, volume, temperature ensemble (NVT; with a modified Berendsen thermostat with velocity rescaling^26^ at 300 K and a 0.1 ps time step; separate heat baths for protein and solvent); the second one in the constant particle number, pressure, temperature ensemble (NPT; Parrinello–Rahman pressure coupling^27,28^ at 1 bar with a compressibility of 4.5 × 10^−5^ bar^−1^ and a 2 ps time constant). In both equilibration runs, a position restraint potential with a force constant of 1000 kJ mol^−1^ was imposed on all protein atoms except hydrogens. For the subsequent MD production runs (588 ns for Pr and 550 ns for Pfr), the same temperature and pressure coupling were used. No position restraints were used, and coordinates were saved every 100 ps.

All bonds to hydrogen atoms were constrained using the linear constrained solver (LINCS)^29^ with an order of four and one iteration. A grid-based neighbor list with a threshold of 10 Å was used and updated every five steps (10 fs). For long-range electrostatic interactions above 10 Å, the particle-mesh Ewald method^30,31^ was used with a fourth-order interpolation and a maximum spacing for the FFT grid of 1.6 Å. Lennard–Jones interactions were cutoff above 10 Å. A long range dispersion correction for energy and pressure was used to compensate for the Lennard–Jones interaction cutoff.^22^

### D. Pairwise comparison analysis

For each MD frame of the Pr and Pfr trajectories, the X-ray scattering was calculated with the software *sastbx*^32^ using a three-dimensional Zernike expansion (options: znk_nmax = 40, n_step = 100) in a *q*-range between 0 and 5 nm^−1^. The difference scattering (Pfr-Pr) curves for all combinations of Pr and Pfr structures from the MD simulation were scored against the experimental data by using the sum of squares of the error (SSE) in the *q*-range between 0.5 and 2.5 nm^−1^. The SSE was determined by minimizing the difference between the experimental and calculated difference scattering against the scaling factor k^10^

$$SSE_{\left(pfr,\;pr\right)}=min_{k}\sum_{q}{\left(\Delta S\left(q\right)-k\times \left(S_{pfr}-S_{pr}\right)\right)}^{2},$$ (1)

where *q* is the modulus of the scattering vector, Δ*S* is the experimental difference scattering, and *S*_pfr_ and *S*_pr_ are the calculated scattering for a given Pfr and Pr structure, respectively.

### E. Fourier transform infrared (FTIR) spectroscopy

The samples were concentrated to an approximate concentration of 2.5 mM with Amicon Ultra Centrifugal Filters (Merck-Millipore). A sample volume of 2 *μ*l was placed on a CaF_2_ window and sandwiched between two window plates using Glisseal grease (Borer Chemie). The resulting sample thickness was approximately 10 *μ*m. The extent of hydration was assessed from the absorption ratio between Amide I (at 1645 cm^−1^) and Amide II (at 1580 cm^−1^) bands, which was about 0.9/0.35 in each sample. The FTIR difference spectra were recorded with a FTIR spectrometer (Nicolet) by utilizing red (λ = 655 nm) and far-red (λ = 785 nm) light-emitting diodes in a consecutive manner. The spot diameters of red (5 mW) and far-red (40 mW) diodes were approximately 1 cm^2^. The samples were illuminated until the photo equilibrium was reached, which took typically several seconds. The baseline was recorded during each switch before changing the illumination conditions. About 20 scans of each state were measured with 2 cm^−1^ spectral resolution. All measurements were done at room temperature.

## III. RESULTS

**FIG. 2.**
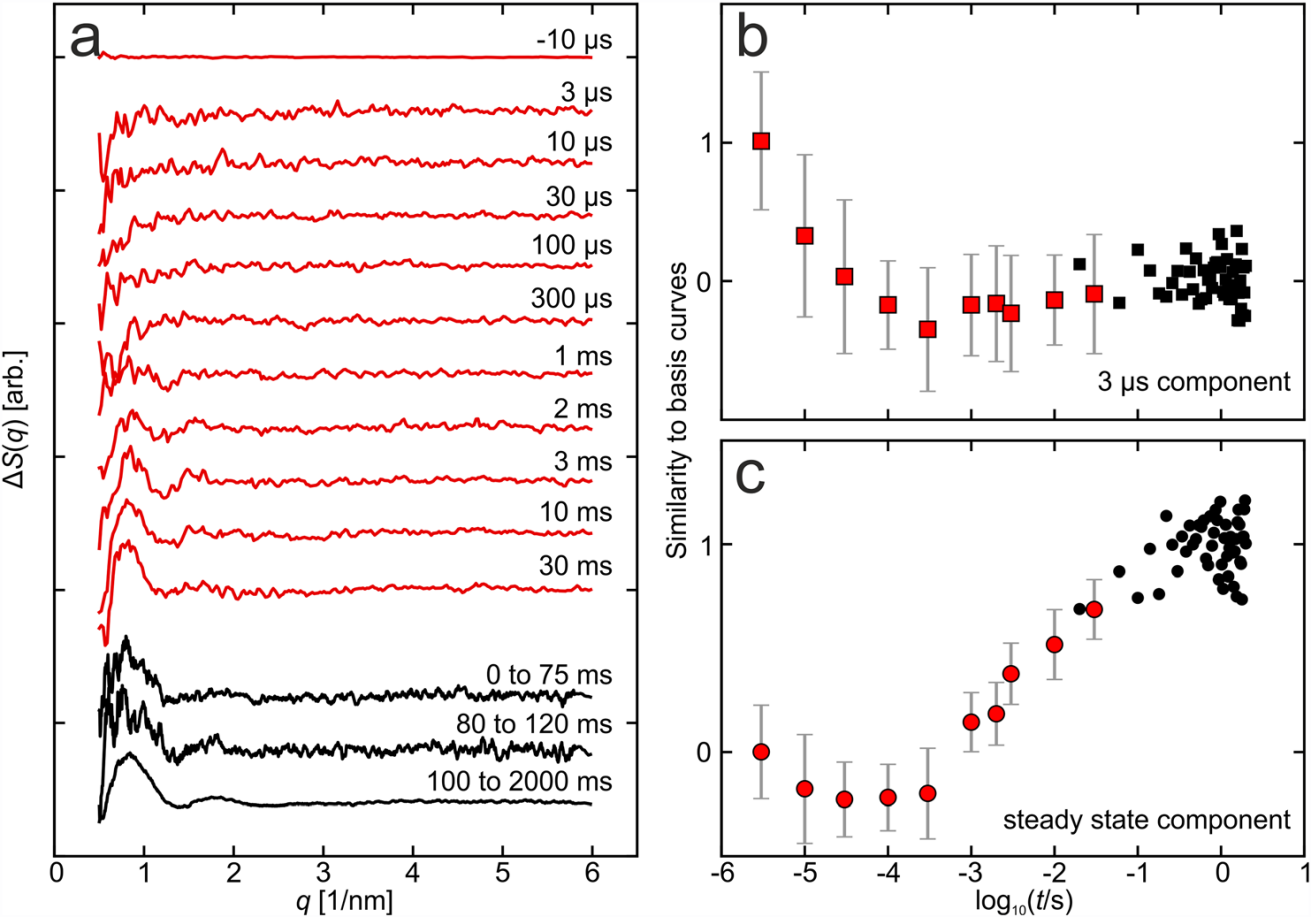
Time-resolved difference X-ray scattering of PAS-GAF-PHY_mon_. (a) Data measured at beamline ID09b at the European Synchrotron Radiation facility (*red*) and data measured at the beamline cSAXS at the Swiss Light Source (*black*) are shown. All data were corrected for minor contributions from heat as described in Ref. 19. ((b) and (c)). Deconvolution of the data into two basis patterns. We used the difference X-ray scattering pattern at 3 *μ*s and the steady state component, measured at 100 ms–2000 ms from (a) to compute the time dependencies shown in panel (b) and (c) by matrix division.

Time-resolved X-ray scattering measurements give information about the structural changes of proteins in a solution.^33,34^ We collected X-ray scattering data of PAS-GAF-PHY_mon_ after illumination with short red-light pulses. The data were referenced to resting state patterns, yielding time-dependent difference X-ray scattering (Fig. 2(a)). On microsecond time scales, PAS-GAF-PHY_mon_ gave no detectable difference signal, except for a dip at the low end of the *q*-range. After completion of the photocycle at *t* > 20 ms, a difference X-ray scattering signal was observed with a positive peak at *q* = 0.8 nm^−1^ and a number of oscillations with smaller amplitude at higher *q* (Fig. 2(a)). These data reflect the difference in X-ray scattering between the Pr and Pfr states.

The peak at *q* = 0.8 nm^−1^ was also observed in the difference between two conventional small-angle X-ray scattering (SAXS) measurements of pre-illuminated Pr and Pfr samples (see Fig. S1(a) in supplementary material).^16^ In the data presented here, the noise level and reliability are greatly improved and oscillations extending to higher *q* are resolved (Fig. 2). Compared to the previously reported difference X-ray scattering of dimeric PAS-GAF-PHY,^10^ the signal on millisecond time scales displays a different peak position and a smaller signal relative to the absolute X-ray scattering (Figs. S1(b) and S2 in supplementary material).

The time-evolution of the data is described by two components, represented by the difference scattering at *t* = 3 *μ*s and *t* > 20 ms. The kinetics of the components are illustrated in Figures 2(b) and 2(c). The first component decays on the microsecond time scale. Its origin is unclear, and we do not discuss it further in this paper. The second signal grows with a half-time of 10 ms. This component reflects the formation of the Pfr state. Its rise is one order of magnitude slower than the rise of the corresponding signal in the dimeric PAS-GAF-PHY (half-time of 1 ms).^10^ We verified that the kinetics and the line shape of the difference scattering are not changed when lowering the protein concentration by a factor of 5. Also, the difference signal was fully reversible (Fig. S2 in supplementary material).

**FIG. 3.**
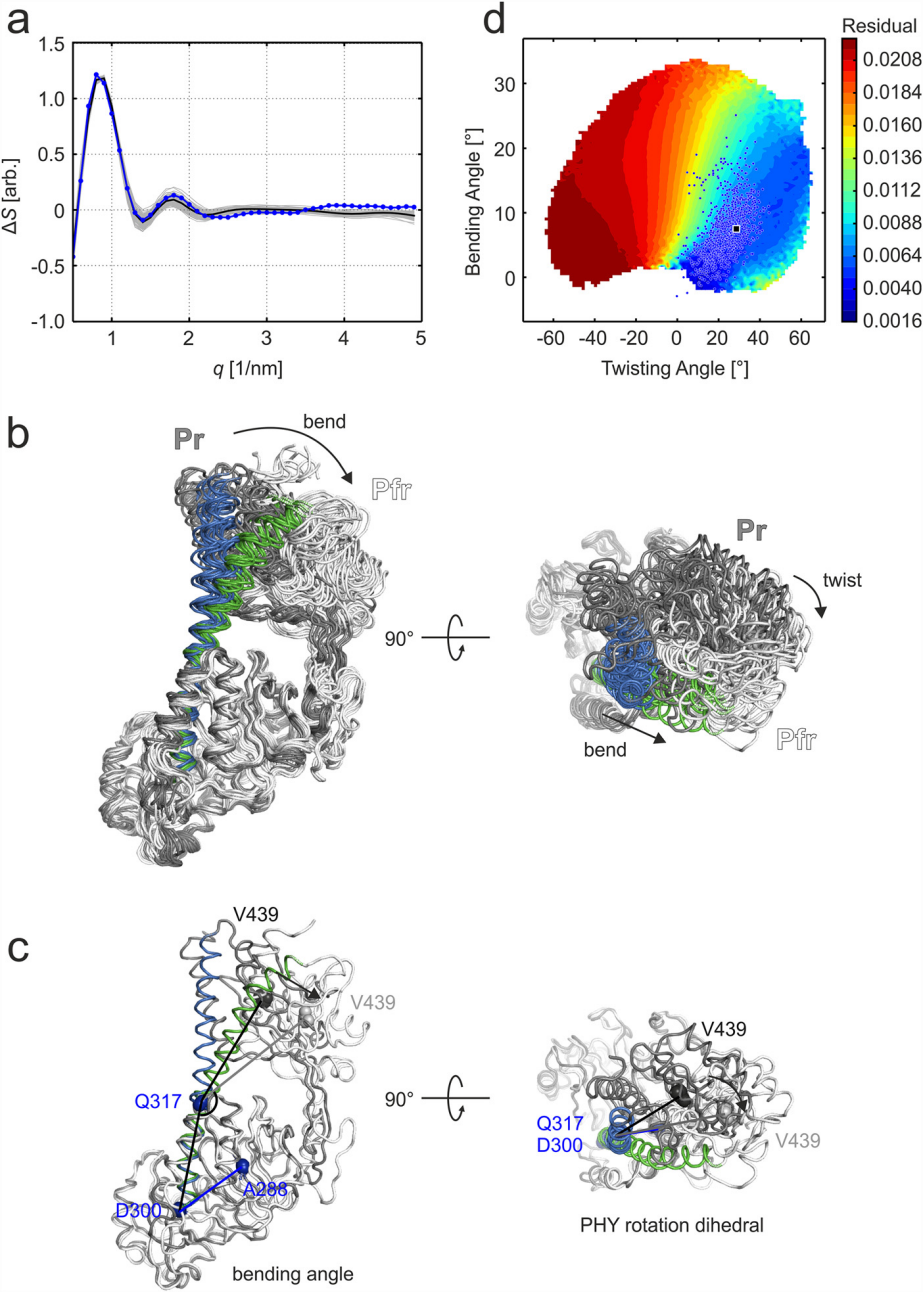
Structural analysis of the difference X-ray scattering for PAS-GAF-PHY_mon_. (a) The “static” (Pfr-Pr) experimental difference X-ray scattering (*blue*) is shown together with the calculated difference scattering for the 1000 Pr/Pfr pairs with the lowest residual (every 10th line is shown in *grey*). The average calculated difference scattering from these pairs are shown as a *black* line. (b) Overlay of the Pr and Pfr structures from the ten Pr/Pfr pairs with lowest residuals. The structures were aligned at their PAS-GAF part. The changes between the Pr (*grey*) and Pfr (*white*) structures reveal a bend of the long scaffolding helix (*blue*/*green*) along with a reorientation of the PHY domain. Also, a twist of the PHY domain relative to the PAS-GAF domains was observed. (c) Bending and dihedral angles chosen for structural analysis of the difference data. The representative Pr and Pfr structures are colored as in panel (b), and the residues selected for the angle and dihedral definitions are marked as spheres. The bend angle was defined by three residues that resided in the start of the long scaffolding helix (D300), in a hinge point of the helix (Q317) and in the center of the PHY domain (V439). The dihedral angle was formed between a reference plane (formed by A288, D300, and E317) and a variable plane (formed by D300, E317, and V439). The residues that reside in PAS-GAF are considered static and are colored as blue; the moving residues are in dark grey (Pr) and pale grey (Pfr). (d) The two modes of motion were chosen to analyze the agreement of all pairs from the structural fitting routine with the data, where the mean residual of the best 10% of fitted pairs are plotted as a function of dihedral and bending angles. The best 1000 structural pairs are plotted in the graph as circles, and the structural pair with the lowest residual is marked with a black rectangle.

The *q* value at which a difference peak occurs gives information about the length scale of the structural changes. The positive peak at *q* = 0.8 nm^−1^ in PAS-GAF-PHY_mon_ indicates a change in a tertiary structure (see curves in Fig. 2(a) at *t* > 20 ms). In order to structurally interpret this difference X-ray scattering, we first created 5888 and 5506 candidate structures by running molecular dynamics simulations in Pr and Pfr, respectively. The simulations started from the Pr and Pfr solution structures of the dimeric PAS-GAF-PHY with one monomer removed.^10^ This collection of structures covers a large subset of the conformational space (Fig. 3), and the molecular dynamics simulations ensure that all structures are realistic. We then computed the difference X-ray scattering for all possible pairs. These difference scattering curves were scored against the experimental X-ray scattering data for *t* > 20 ms in Eq. (1). The difference scattering signal from the best 1000 structural pairs shows excellent agreement with the experiment (Fig. 3(a)) within the *q* range of 0.5–2.5 nm^−1^. This scattering region gives information about the large-scale movements of the protein. The small structural features indicated by the higher scattering angles (*q* > 2.5 nm^−1^), however, were not sampled by the simulations.

Figure 3(b) illustrates the structures of the Pr and Pfr states that participated in the 1000 best fitting pairs. Inspection of the structure reveals two components. First, the monomer bends along the long scaffolding helix (side view, “bend”). Second, the PHY domains twist with respect to the chromophore binding domains (top view, “twist”). The same types of structural changes were observed for all of the Pr and Pfr states that participated in the pairs with the lowest scores.

In order to quantify these observations, we chose two representative angles (Fig. 3(c)). The bending angle was defined to be between one of the ends of the long scaffolding helix (Asp300), a hinge point of the helix (Gln317), and the center of the PHY domain (Val439). The dihedral angle was defined between Ala288, Asp300, Gln317, and Val439. As the next step, all Pr/Pfr pairs were binned according to their changes in dihedral and bending angle. The average of the lowest 10% residuals in each bin was plotted in (Fig. 3(d)). The contour plot reveals a broad minimum at around 5° and 20° for a change in bending and dihedral angle, respectively. The twisting and bending angles of the best fitting pairs (circles in Fig. 3(d)) also group around this minimum. This demonstrates that a structural change along both coordinates is robustly encoded in the data.

We note that light-dependent oligomerization is unlikely to be the reason for the observed difference scattering signal. First, analytical gel filtration analyses indicated that the samples are purely monomeric.^16^ Second, the difference scattering appears to approach 0 at *q* = 0. Third, Fig. S1(a) shows that the reported difference scattering signal agrees with the difference of a SAXS measurement recorded for PAS-GAF-PHY_mon_ in Pr and Pfr.^16^ From the latter SAXS measurements, it had been found that scattering at *q* = 0, or I(0), is essentially the same in Pr and Pfr, which is a clear indication that the oligomeric state of the protein does not change upon illumination.

**FIG. 4.**
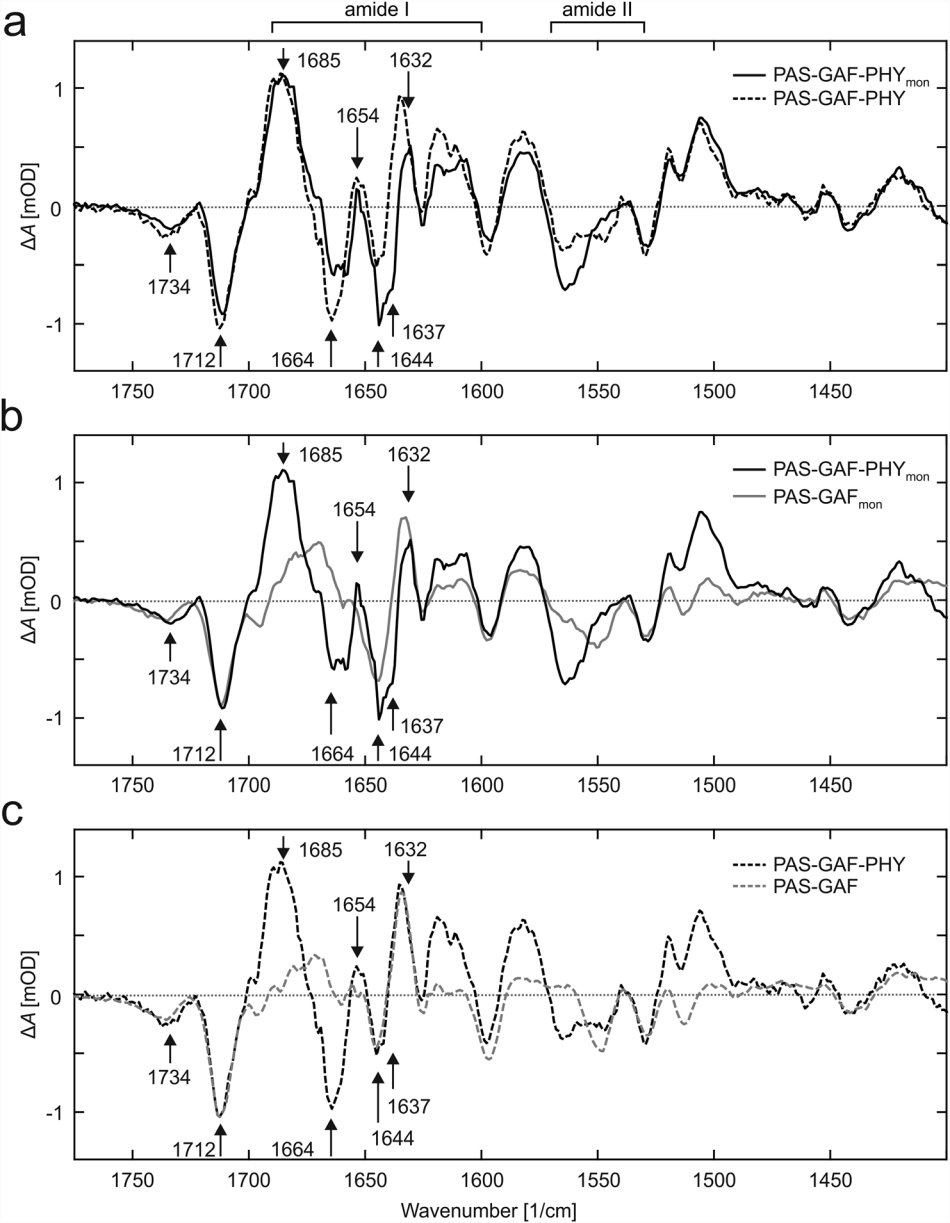
Infrared absorption spectra indicate a change of fold in the PHY tongue. (a) The FTIR difference spectra (Pfr-Pr) of PAS-GAF-PHY and PAS-GAF-PHY_mon_ are similar except for the spectral regions around 1550 cm^−1^ (amide II) and from 1600 cm^−1^ to 1690 cm^−1^ (amide I), which show some deviations. A positive signal 1654(+) cm^−1^ represents the increase of the helical content of the protein. The negative feature at 1637 cm^−1^ indicates a reduced β sheet content of the protein and is present in both samples but is poorly resolved in wild-type PAS-GAF-PHY. (b) When compared to PAS-GAF_mon_, the difference spectra of PAS-GAF-PHY_mon_ show the negative feature at 1637 cm^−1^. This feature is partially masked by the signals 1644(−) cm^−1^ and 1632(+) cm^−1^ that originate from the changes in the PAS-GAF region. (c) Comparison between the dimeric PAS-GAF-PHY and PAS-GAF fragments shows features similar to their monomeric counterparts in panel B. The 1654(+) cm^−1^ signal is present in the PAS-GAF-PHY, whereas the negative signal at 1637 cm^−1^ is poorly resolved. The data in panel A are scaled to the 1685 cm^−1^ peak, and the data in B and C to the 1712 cm^−1^ peak. The peak positions in all panels are quoted for PAS-GAF-PHY_mon_.

In a previous study, we have proposed that the structural signal in phytochromes is transduced by refolding of the PHY tongue.^10^ Characteristic peaks in vibrational spectra of this transition have been identified.^9,35^ We performed Fourier transform infrared (FTIR) spectroscopy measurements on PAS-GAF-PHY_mon_, PAS-GAF_mon_, and their dimeric counterparts (Fig. 4). The difference FTIR spectra (Pfr-Pr) show high agreement between the PAS-GAF-PHY samples, except for the amide I (1600–1690 cm^−1^) and amide II (1530–1570 cm^−1^) spectral regions (Fig. 4(a)).

The spectral signatures of the carbonyl stretching vibrations at 1734(−) cm^−1^ and 1712(−) cm^−1^ stem from the chromophore^9,35,36^ and were similar in all constructs reported here (Fig. 4). This demonstrates that all investigated fragments undergo similar structural changes in and around the biliverdin. The signal at 1685(+) cm^−1^ is present in PAS-GAF-PHY and PAS-GAF-PHY_mon_, but not in PAS-GAF fragment. We therefore conclude that the signal stems from the PHY domain. Amide I signals characteristic for changes in the content of β-sheets and α-helices were found at 1637(−) cm^−1^ and 1654(+) cm^−1^, respectively (Fig. 4). We assign these features to refolding of the PHY tongue, which have been previously detected by X-ray crystallography^10^ and infrared spectroscopy of other phytochromes.^9,35^ The assignment is supported by that the spectral features are present in both photosensory core fragments (PAS-GAF-PHY) but not in PAS-GAF lacking the PHY tongue. The peaks are more prominent in PAS-GAF-PHY_mon_. This is because the PAS-GAF dimer peaks at ca. 1632(+) cm^−1^ and 1644(−) cm^−1^ partially mask the 1637(−) cm^−1^ signal in PAS-GAF-PHY (Figs. 4(b) and 4(c)). The peaks related to PAS-GAF variants and the differences between the samples in the amide II region remain unassigned. Nevertheless, the FTIR data are consistent with the PHY tongue refolding in the dimeric and monomeric variant of PAS-GAF-PHY.

## IV. DISCUSSION

The dimeric photosensory core fragment of the phytochrome from *D. radiodurans* (PAS-GAF-PHY) was previously found to undergo a large structural rearrangement, which increases the PHY-PHY distance.^10^ Here, we find that the same type of rearrangement is also present in the monomeric photosensory core (PAS-GAF-PHY_mon_). Compared to the previous study, the new data reveal more structural details. The dominant structural changes are a bend and a twist of the PHY domains with respect to the PAS-GAF domains (Fig. 3(b)). Similar rearrangements have also been observed with cryo-electron microscopy of the full-length phytochrome.^37,38^ The electron micrographs indicated a light-induced separation (bend) and re-orientation (twist) of the sister PHY domains in the full-length phytochrome.

It is intuitive that the bend and twist movements are a consequence of the same structural rearrangement that is caused by the shortening of PHY tongue in the Pr-to-Pfr transition.^10^ The infrared spectroscopy data reported in this paper show that the dimeric and monomeric photosensory core fragments undergo refolding of the PHY tongue in a solution (Fig. 4). Although the FTIR spectra from different phytochrome species vary, the same refolding signature has been observed in BphP2 from *Rhodopseudomonas palustris*,^9^ Agp1 from *Agrobacterium tumefaciens*,^39^ and Cph1 from *Synechocystis* PCC 6803.^40^

The FTIR data further demonstrate that the structural and chemical changes of the PAS-GAF domains are very similar between monomeric and dimeric counterparts. Only small differences are observed in the Amide I and Amide II regions. It is interesting to note that the monomeric PAS-GAF gives stronger difference spectra in the visible spectral region than the dimer.^15,16^

Combining these findings implies that one monomeric photosensory core can be activated structurally even when separated from its dimerization partner. The monomer of the photosensory core is therefore the smallest unit to initiate the structural machinery of the entire phytochrome. It is currently unclear how the two monomer units in a phytochrome dimer work together to activate the output domains. Our data show that the build-up of the final state is slower by approximately an order of magnitude in the monomer mutant than in the dimer. Thus, the dimerization partners have an influence on the kinetics of the light-induced structural turnover. Highly similar dimerization interfaces of the Pr- and Pfr-state crystal structures^10,14,38^ suggest that the two photosensory cores in a dimer can be activated independently of each other. Therefore, this observation indicates that cooperativity is likely restricted to the output domains.

This conclusion also implies that the photosensory core of bacterial phytochrome likely does not require any specific quaternary arrangement for its activation. Different dimer arrangements have been found among phytochromes. For example, fragments of *Dr*BphP, *Rp*BphP2, *Pa*BphP, *At*PhyB crystallize as parallel dimers,^10,12,38,41,42^ whereas Cph1, Cph2, *Rp*BphP1, *Rp*BphP3 crystallize as antiparallel dimers.^8,11,42,43^ SAXS and electron microscopy also support modes of dimerization, where the photosensory core domains do not interact and only the output domains form the dimerization contacts.^44,45^ In many bacterial phytochromes, such as the phytochrome studied here, parallel dimer organization seems to be predominant.^37,38^ It could be that different dimer arrangements of different phytochromes lead to different modes of action in the output domains. However, the conformational changes in the monomeric photosensory core module, which we study here, are independent of this arrangement.

The photosensory module of phytochromes appears to be structurally highly conserved. Conventional phytochromes have a PAS-GAF-PHY domain arrangement with a long scaffolding helix and a PHY tongue extension. Therefore, we propose that the structural changes identified here are likely widely conserved in plants, fungi, and bacteria.

## SUPPLEMENTARY MATERIAL

See supplementary material for supplementary Figures 1 and 2.
